# Animal Use Strategies in the Longshan Mountain Region of Northern China during the First Millennium BC: A Zooarchaeological Analysis of Yucun

**DOI:** 10.3390/ani13243765

**Published:** 2023-12-06

**Authors:** Tianyu Zong, Borui Du, Chengrui Zhang, Feng Sun, Zexian Huang, Ruoxin Cheng, Kexin Liu, Tao Shui, Yongan Wang, Yue Li

**Affiliations:** 1 School of Cultural Heritage, Northwest University, Xi’an 710127, China; 2 Institute of Archaeology, Chinese Academy of Social Sciences, Beijing 100101, China; 3 Department of Anthropology, Harvard University, Cambridge, MA 02138, USA; 4 Gansu Provincial Institute of Cultural Relics and Archaeology, Lanzhou 730015, China; 5 School of History, Nanjing University, Nanjing 210023, China; 6 China-Central Asia “the Belt and Road” Joint Laboratory on Human and Environment Research, School of Cultural Heritage, Northwest University, Xi’an 710127, China; 7 Key Laboratory of Cultural Heritage Research and Conservation, Northwest University, Xi’an 710127, China

**Keywords:** China, Bronze Age, the Longshan Mountain region, zooarchaeology, subsistence economy, environmental conditions

## Abstract

**Simple Summary:**

Through a preliminary analysis of animal remains from the large settlement site of Yucun, located east of the Longshan Mountain and associated with the Zhou people during the first half of the first millennium BC, the authors investigated the subsistence practices in relation to the exploitation of animal resources by the site’s residents. The examination of animal species representation and the mortality profiles of major domesticates suggest similarities between Yucun and sites associated with the Qin people, located to the west of the Longshan Mountain. However, differences were observed when comparing Yucun to other contemporaneous sites in the middle and lower Yellow River valley. These patterns in animal use strategies appear to have been influenced by local environmental conditions.

**Abstract:**

The first millennium BC saw the expansion of the Western Zhou dynasty in its northwestern frontier, alongside the rise and development of the Qin State in the Longshan Mountain region of northern China. Exploring the subsistence practices of these communities is crucial to gaining a better understanding of the social, cultural, and political landscape in this region at the time. While much of the research to date has focused on the Qin people, the subsistence practices of the Zhou people remain poorly understood. In this study, we analyzed animal remains from Yucun, a large settlement site associated with the Zhou people, located to the east of the Longshan Mountain. These animal remains were recovered in the excavation seasons of 2018–2020. Our results show that pigs, dogs, cattle, caprines, and horses, which were the major domestic animals at Yucun, accounted for over 90.8% of the animal remains examined in terms of the number of identified specimens (NISP) and 72.8% in terms of the minimum number of individuals (MNI), with cattle and caprines playing dominant roles. In terms of the taxonomic composition and the mortality profiles of pigs, caprines, and cattle, Yucun shared similarities with Maojiaping and Xishan, two contemporaneous Qin cultural sites located to the west of the Longshan Mountain, and differ from other farming societies in the middle and lower reaches of the Yellow River valley. Considering the cultural attributes and topographic conditions of these various sites, these findings imply that environmental conditions may have played a more significant role than cultural factors in shaping the animal-related subsistence practices in northern China during the first millennium BC.

## 1. Introduction

Animal resources play a pivotal role among the material assets within human societies. Animals not only provide essential food and energy for human sustenance, but also serve as sources of diverse products crucial to social development [1,2]. This multi-faceted role of animals highlights their significance as an indispensable component of human civilization [3,4,5]. Through the examination of animal resource acquisition and exploitation, the exploration of subsistence economics offers a fresh perspective that facilitates our understanding of the intricate relations between ancient communities, the land they resided, social advancement, and cultural transmission [6,7,8]. Moreover, it provides insights into the origins and evolution of civilization and the driving forces behind it [2,9,10,11].

Zooarchaeological work in Northwestern China has significantly contributed to our understanding of the economic and cultural transformations during the second and first millennia BC. This crucial period marked the alternation of Bronze Age states and the accumulation of resources that eventually led to the establishment of the first united empires in China [10,12]. The Western Zhou period (ca. 1046-771 BC), in particular, witnessed the formation of political institutions and ritual systems that had profound influences on the following era. The gradual decline of the Western Zhou authority set the stage for the rise of another community in its west, the Qin people, who formed the Qin State and eventually the Qin Empire by the end of third century BC [13,14]. During this critical period, the Longshan Mountain region emerged as a crucial focal point, where different farming populations and pastoralists engaged in economic, social, and military interactions [15,16].

Situated at the crossroads of today’s Shaanxi and Gansu provinces and the Ningxia Hui Autonomous Region, the Longshan Mountain has long acted as a natural barrier, separating the Central Plains from the northwest. As the northwestern frontier of the Western Zhou dynasty, the Longshan Mountain region witnessed the expansion of the Zhou people and the rise and development of the Qin State during the first millennium BC [16,17,18,19]. The region also served as a stage for interactions between the agricultural Zhou and Qin people and the pastoral Rong people, as evidenced by the intricate cultural characteristics in the eastern and western flanks of the Longshan Mountain [20]. Therefore, an in-depth examination of the subsistence economies of these communities is imperative for a more comprehensive understanding of the economic, social, and political developments in Bronze Age northern China.

Traditionally, research on the subsistence practices in the Longshan Mountain region during the first millennium BC has centered on the ancient *Bin* area [21]. Located in the Jing and Wei River valleys to the east of the Longshan Mountain, this area played a significant role in the political transformation and the rise of the Zhou people in the late second millennium BC [22]. While much attention has been given to the pre-Western Zhou period, there remains a dearth of research on the subsequent expansion and decline phases of the Zhou people [23]. Despite an increase in the number of studies on the Eastern Zhou period, much of the research has prioritized the Qin and Rong societies, exemplified by the zooarchaeological investigations of Maojiaping and Xishan [24,25]. Many questions remain about how the Zhou people in the Longshan Mountain region sustained themselves through subsistence practices during this period, and how these actions compare with those employed by contemporaneous sites associated with other societies.

Here, we report the results of a zooarchaeological study of Yucun, a site located to the east of the Longshan Mountain. We examined the species representation of the animal assemblages recovered, analyzed the mortality profiles for the major animal domesticates, and compared the datasets with those from several other sites located to the west of the Longshan Mountain. The results provide evidence for the animal use strategies of the Zhou people who established settlements for survival following the collapse of the Western Zhou dynasty in the early first millennium BC. Our findings shed light on the nuanced dynamics of interaction between the Zhou people and their neighboring societies during this critical period.

## 2. Materials and Methods

### 2.1. The Site of Yucun and the Animal Remains Examined

**Figure 1:**
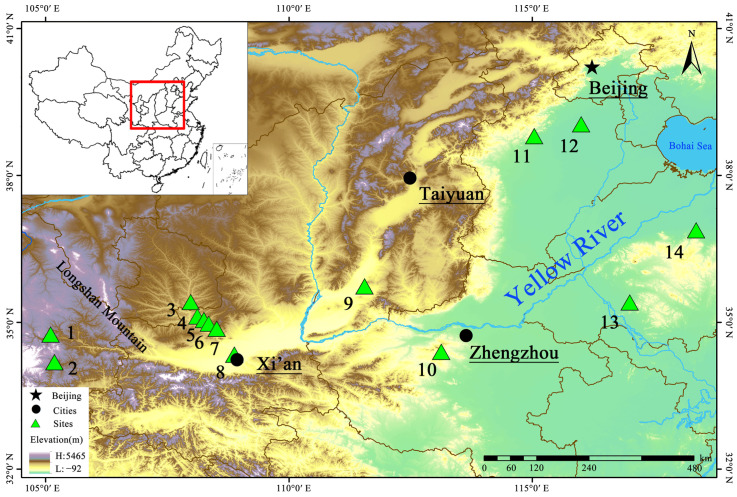
Location of archaeological sites mentioned in this study: (1) Maojiaping; (2) Xishan; (3) Yucun; (4) Xitou; (5) Sunjia; (6) Zaolinhetan; (7) Zaoshugounao; (8) Gongbeiya; (9) Tianma-Qucun; (10) Wangchenggang; (11) Nanfangshui; (12) Nanyang; (13) Zhuguogucheng; (14) Kanjiazhai.

Yucun is located on the Zaosheng loess terrace to the east of the Longshan Mountain in the present-day Ningxian County of the eastern Gansu Province (Figure 1). Archaeological surveys and excavations from 2018 to 2020, directed by the Gansu Provincial Institute of Cultural Relics and Archaeology and the School of History, Nanjing University, suggest that Yucun was a large settlement site [26].

**Figure 2:**
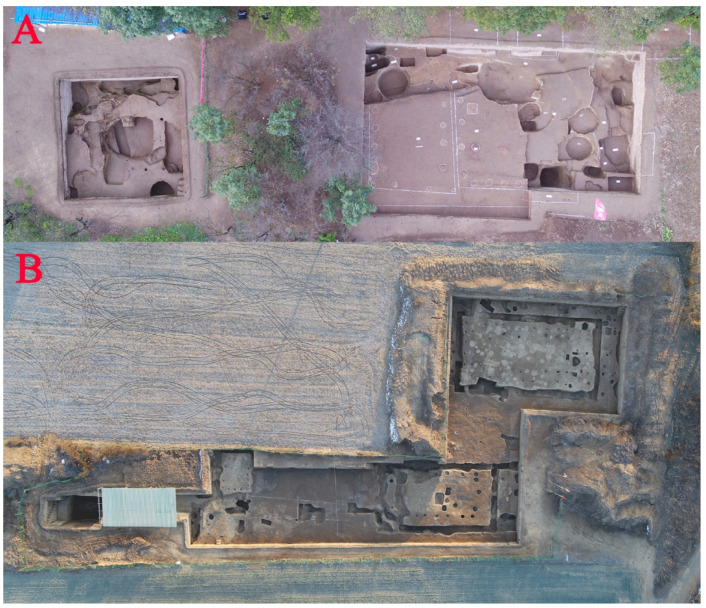
Photos showing different excavation units at Yucun: (**A**) Excavation area in 2020. (**B**) Excavation area in 2019 and 2020. Photos taken by Yongan Wang.

Within an excavated area of 3100 m^2^, a walled and moated enclosure was uncovered. This enclosure had a roughly rectangular shape from east to west, with the southwestern section partially damaged by a modern ditch. Within the walls, archaeological features including architectural foundations, pits, ditches, roads, kilns, and reservoirs were found (Figure 2). In addition, several burials and chariot–horse pits were discovered outside the enclosure [27]. The excavations yielded a substantial number of artifacts, including ceramics, lithics, bones, bronze, and jade artifacts, and animal remains. According to the style of artifact assemblages and the stratigraphic relations, the walled settlement at Yucun was dated to the late Western Zhou to the early to mid-spring and -autumn periods (i.e., the first half of the first millennium BC) [28]. As a large settlement discovered for the first time from this period in the region east of the Longshan Mountain, Yucun offers new archaeological material for investigating the social and cultural exchanges in the region and beyond during the first millennium BC. It also holds importance in understanding the evolution of the Zhou people from their rise to decline [27].

In this research, we analyzed a total of 6686 animal specimens recovered from 174 contexts, including 3 strata, 119 pits, 31 ditches, 9 houses, 3 reservoirs, 2 pillar holes, 2 roads, 1 kiln, 1 storage pit, and 3 burials (The animal remains were recovered from the infilling of these burials). These animal remains were hand-collected during excavation, with dry sieving employed in nearly all contexts.

### 2.2. Methods

The zooarchaeological analysis was conducted on-site. We recorded the taphonomic effects to evaluate the preservation conditions of the animal remains examined. The identification of animal species referred to the published atlases of skeletal elements [29,30,31] and the archaeological and modern animal specimens stored as teaching resources in the Zooarchaeology Laboratory of the School of Cultural Heritage, Northwest University.

Each piece of the animal remains was identified at the lowest taxonomic level. The indeterminate specimens, such as rib fragments, were identified as large, medium, and small mammals according to the size classes. The animal remains identified as Osteichthyes, birds, rodents, large Artiodactyla, and mammals of the family level were included in calculating the number of identified specimens (NISP) and the minimum number of individuals (MNI). The specimens identified as sheep (*Ovis aries*), goat (*Capra hircus*), and sheep/goat (*Ovis aries*/*Capra hircus*) were grouped into one category as “caprines”, while the specimens identified as sika deer, roe deer, and large/small cervids were categorized as “cervids” in the discussion section.

The mortality profiles for the pigs and caprines were reconstructed based on dental eruption and wear [32]. The correspondence between the wear stages and ages followed the studies of Li [33,34]. If the age estimates for a given specimen covered multiple age groups, this specimen was divided equally and counted into each group. The analysis of the cattle age was based on bone epiphyseal fusion [35].

## 3. Results

### 3.1. Post-Depositional Effects

Weathering was observed in a small proportion (5.2%) of the animal remains examined, while rodent gnawing and carnivore chewing were identified in 3.1% and 2.0% of the samples, respectively. Approximately 2.1% of the skeletal elements displayed traces of burning. In general, post-depositional effects had minimal impact on the animal remains from Yucun.

### 3.2. Traces of Human Modification

Evident traces of human modification were identified in 358 animal remains, constituting 5.4% of the entire assemblage analyzed. The most prevalent modifications observed were saw marks, present on 202 skeletal elements, followed by polish marks (104 skeletal elements) and cut/incision marks (39 skeletal elements). Specifically, cut/incision marks were primarily seen on the limb bone shafts and the ventral part of the atlas. Other human-induced marks include chisel marks, rasp marks, and drill marks.

### 3.3. Animal Taxonomic Structure

**Figure 3:**
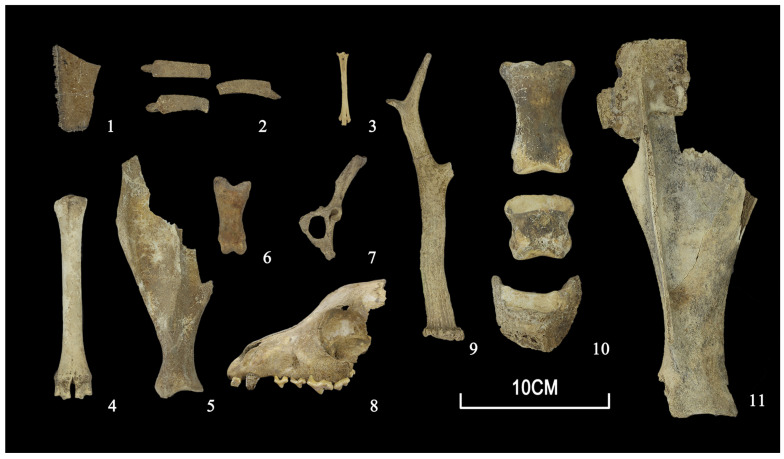
Typical animal specimens from Yucun: (1) Geoemydidae, plastron; (2) Trionychidae, carapace; (3) *Phasianus* sp., tarsometatarsus; (4) *Ovis aries*, metatarsal; (5) *Sus domesticus*, humerus; (6) *Ursus thibetanus*, first phalanx; (7) *Lepus* sp., pelvis; (8) *Canis familiaris,* skull; (9) *Capreolus capreolus*, antler; (10) *Equus caballus*, second, third, and fourth phalanges; (11) *Bos taurus*, scapula.

The animal assemblages from Yucun consisted of Lamellibranchia, Osteichthyes, Aves, Reptilia, and Mammalia (Figure 3), with mammalian remains (97.9%) constituting the largest proportion. The NISP of the animal remains is 4521, representing a minimum of 243 individuals (MNI) (Table 1).

Domestic animals, including pigs, dogs, cattle, caprines, and horses, predominated the animal remains examined. By the NISP, cattle (1225/27.1%) were the most common domestic animals, followed by caprines (1134/25.1%), pigs (917/20.3%), horses (464/10.3%), and dogs (365/8.1%). The MNI structure was similar to that of the NISP, except that pigs (45/18.5%) and dogs (35/14.4%) took up larger proportions. As for wild animals, cervids occupied the largest part in terms of both the NISP (211/4.7%) and MNI (24/9.9%).

### 3.4. Mortality Profiles for the Major Domesticates

#### 3.4.1. Pigs

**Figure 4:**
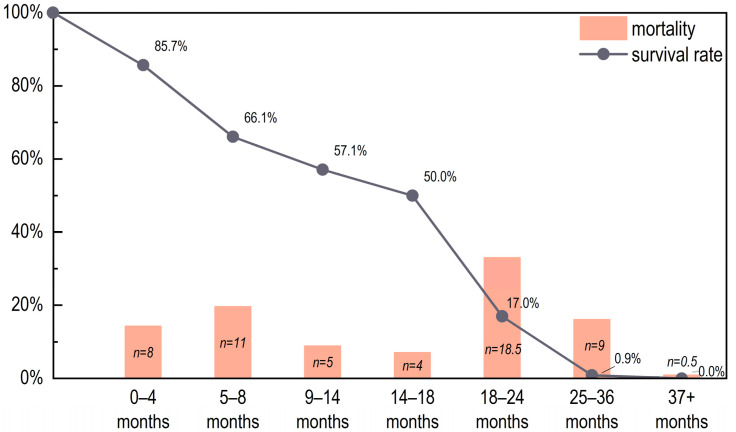
The mortality profile and survivorship curve for the pigs from Yucun.

Fifty-six right mandibles were used to reconstruct the pig mortality profile at Yucun. The results show that the largest kill-off occurred at 18–24 months, with more than one third of the individuals being culled at this stage. This was followed by the stages of 25–36 months (19.6%) and 5–8 months (16.1%). Less than 1.0% of the pigs died at ages older than 3 years old (Figure 4). Normally, the optimal slaughtering age for pigs is 1–2 years of age, with large numbers of pigs killed for pork, and only a few individuals kept for breeding [36]. The majority (over four fifths) of the pigs did not survive beyond 2 years of age, of which nearly half were killed between 1–2 years of age. This mortality profile implies that the pigs were raised primarily for pork at Yucun.

#### 3.4.2. Caprines

**Figure 5:**
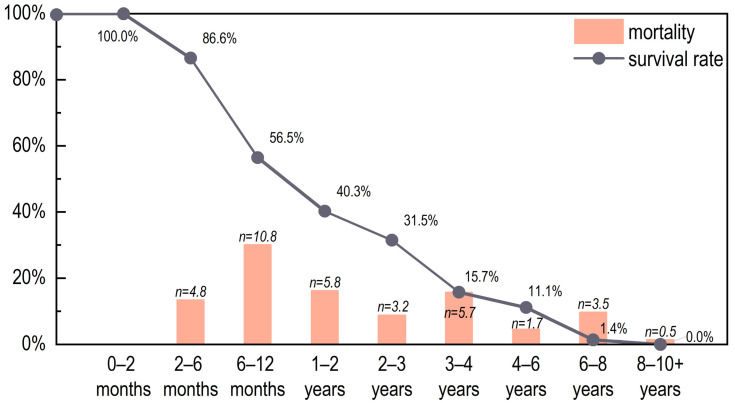
The mortality profile and survivorship curve for the caprines from Yucun.

In total, 36 left mandibles were used to reconstruct the mortality profile of the caprines at Yucun. Caprine slaughtering took place at all age stages except for 0–2 months, with the largest proportion of the caprines dying at an age of 6–12 months (30.1%) (Figure 5). In particular, there was a significant focus on individuals under 4 years of age, which is similar to the mortality profile indicative of meat production, as proposed by Payne [37]. This age structure also mirrors that of the caprine remains found in the Xiaomintun Locale at Anyang, the capital of the Shang dynasty in the late second millennium BC, which aligns broadly with the typical model for meat consumption [34]. The presence of human cut marks at both the proximal and distal ends of the limb bones in some of the caprines further supports this interpretation, as does the presence of human cut marks associated with dismembering or flesh removal recorded in a previous ethnographic survey on modern pastoralists in eastern Xinjiang [38]. These lines of evidence suggest that the primary purpose of caprine herding at Yucun was meat acquisition.

It is also worth mentioning that a large quantity of caprines were killed between 6–12 months of age, which reflects a desire for tender meat. Indeed, the consumption of lambs was recorded in the historical texts, such as *Zhouli* (Rites of Zhou) and *Shiji* (Records of the Grand Historian), and it was a common practice to serve mutton at feasts during this period. Previous research also supports the idea that the killing of lambs at Anyang was possibly for obtaining tender meat or for preparing for harsh seasons [34].

#### 3.4.3. Cattle

The reconstruction of the cattle mortality profile at Yucun relied on the epiphyseal fusion data obtained from a total of 353 cattle skeletal elements. According to our findings (Table 2), all individuals survived beyond the age of 7–10 months, of which most (98.3%) survived beyond 12–18 months of age. However, a noticeable shift in the mortality pattern occurred at 2–3 years of age, with approximately 20.6% of cattle being culled at this stage. More than two thirds of the cattle were not culled until they reached 3–4 years of age. While research has shown that in modern societies beef cattle are normally culled around 1.5–2 years of age for optimal yield [39], it is important to take into consideration that the Bronze Age breeding technologies may have resulted in a different optimal age. An ethnographic survey among modern pastoralists in Inner Mongolia in northern China reveals that cattle aged between 3 and 4 years are often slaughtered for meat [40]. Human cut marks on the joints of the skeletal elements (e.g., radius, tibia, and metacarpal) provide additional evidence that the cattle killed at the age of 2–4 years were primarily raised for meat.

**Figure 6:**
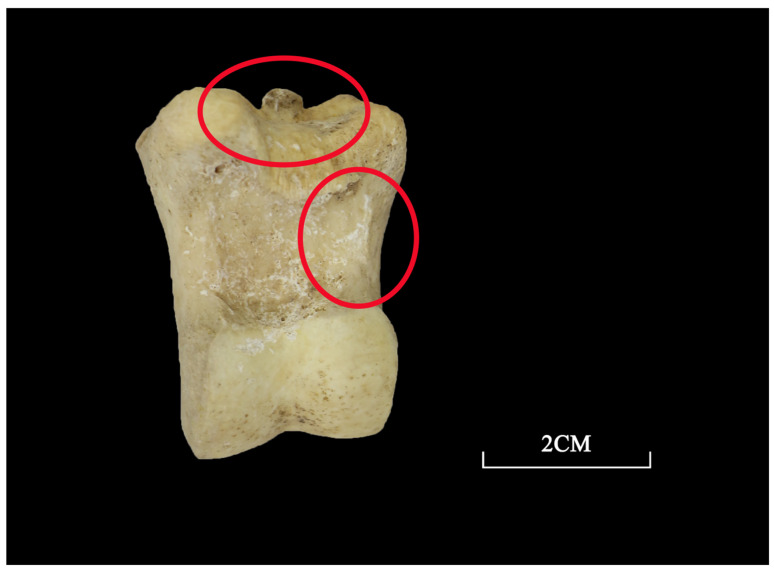
An example of abnormal second phalange of cattle from Yucun.

In addition, over 70% of the cattle examined survived beyond the age of 4 years, a survival rate likely associated with the exploitation of the cattle for labor power. Notably, more than 15.0% of the first or second phalanges of the cattle examined exhibited abnormalities, accounting for over 95% of all the skeletal abnormalities observed in cattle from Yucun (Figure 6). The presence of exostosis in the distal lower limb bones suggests a potential association with draft use [41,42]. Previous research indicates that cattle were indeed used for traction during the Shang period [43]. Taken together, the pathological evidence from Yucun implies that some cattle may have been used for traction purposes to some extent. That being said, this does not exclude the possibility that the pathologies may also be linked to factors such as ageing or genetic predisposition.

## 4. Discussion

The prevalence of domestic animals (90.8% by NISP, 72.8% by MNI) at Yucun indicates a strong reliance on animal husbandry, supplemented by the acquisition of wild species. These strategies for animal resource exploitation provided a robust material foundation for the maintenance of the Yucun community.

To gain a better understanding of the use of animals for subsistence purposes in the Longshan Mountain region during the first millennium BC, we compared the zooarchaeological data from Yucun with those from two contemporaneous sites, Maojiaping (MJP) [24] and Xishan (XS) [25] (Figure 1), both located to the west of Longshan Mountain and associated with the Qin people. Given that the published data from XS solely come from mammals and that the NISP data also include animal remains from sacrificial contexts, which could not be separated, our analysis primarily centered on mammal remains found the in residential contexts of MJP and XS and exclusively used the MNI data.

**Figure 7:**
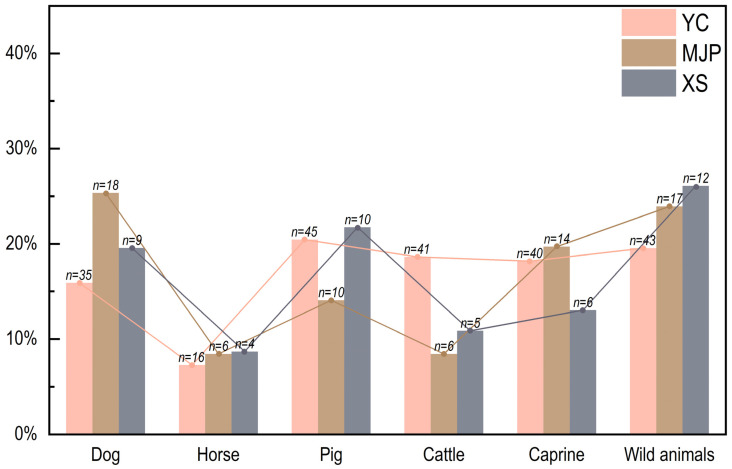
The composition of domestic animals (by MNI) at Yucun (YC), MJP, and XS.

Across the three sites in the Longshan Mountain region, domestic animals consisted of pigs, dogs, cattle, caprines, and horses, with the average proportion of domestic species nearing 80% according to the MNI (Figure 7). A chi-square test on the MNI data indicates that there were no statistically significant differences across these sites (χ^2^ = 9.101, df = 8, *p* > 0.05). This further underscores the overall similarity in the proportion of domestic animals present in the animal assemblages from these sites.

During the first millennium BC, the terraces on the Loess Plateau in the Jing River valley, where Yucun is situated, featured a more favorable ecological environment compared to the present day, exhibiting a higher degree of tree coverage and a more extensive distribution of forests [44,45]. The sites of MJP and XS sit on the loess terrace along the Wei River and the Xihanshui River, respectively. Although these three sites are located on opposite sides of the Longshan Mountain, the entire region shared a common temperate continental monsoon climate. The annual average temperatures and precipitation levels, as well as the topography and geomorphology, were similar [46,47,48]. While Yucun, MJP, and XS were linked to different cultural groups, the parallels observed in animal resource exploitation observed across the three sites may be attributed to the comparable environmental conditions prevalent in the Longshan Mountain region during that era.

The stable isotopic values of humans from Yucun are currently unavailable. However, the data from MJP and XS indicate that during the Bronze Age, the dietary patterns of most residents of the Longshan Mountain region were characterized by the consumption of animal protein, with only a small percentage of people primarily relying on plant-based food [49]. Furthermore, it appears that the subsistence economy at Yucun, located in the upper Jing River valley was similar to that of the sites of Zaoshugounao, Zaolinhetan, Sunjia, and Xitou in the middle Jing River valley, dated to the late second millennium BC, where intensive crop cultivation and pig husbandry, alongside extensive caprine herding, were observed in the archaeological record [50,51,52]. Considering that the landscapes surrounding these sites feature loess terraces and gullies, the geographic and environmental conditions may have contributed greatly to the similarities in the exploitation strategies for both animal and plant resources across these sites.

**Figure 8:**
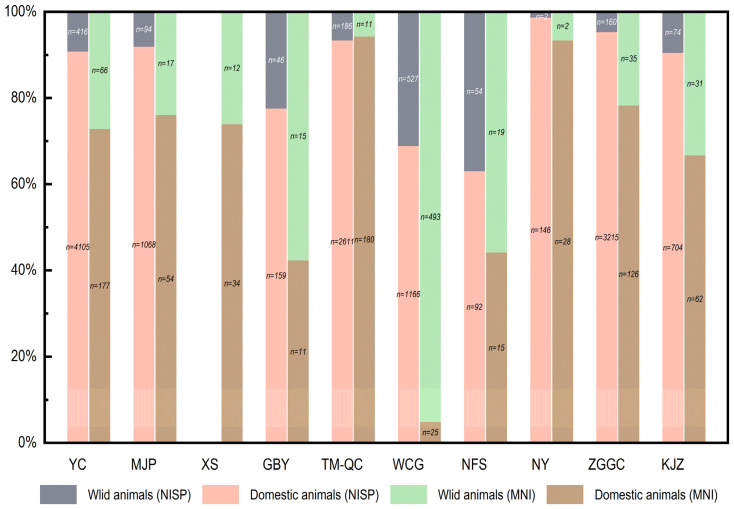
The proportions of domestic and wild animals at different sites mentioned in this study.

During the same period, residents at contemporaneous sites in the middle and lower reaches of the Yellow River, including, but not limited to, Gongbeiya (GBY) [53], Tianma-Qucun (TM-QC) [54], Wangchenggang (WCG) [55], Nanfangshui (NFS) [56], Nanyang (NY) [57], Zhuguogucheng (ZGGC) [58] and Kanjiazhai (KJZ) [59], similarly placed significant emphasis on domestic animals in their subsistence practices (Figure 8). In fact, the average percentage of domestic animals at these sites during the first millennium BC was nearly 88% by NISP. The MNI data were similar. The higher proportion of wild animals according to the MNI at GBY and NFS was likely due to the small number of specimens. In the case of WCG, the apparently low proportion of domestic animals was probably a bias caused by the count of 481 snails (463 identifed to *Cipangopaludina cahayensis* and 18 identified to Fruticicolidae). If these mollusks were excluded, domestic animals would take up approximately 68% of the assemblage in terms of MNI.

In a previous study on the agropastoral economy in ancient Mesopotamia and Anatolia, the ratio of caprine to pig and cattle, alongside the ratio of wild seeds to cereals, were used to infer extensive or intensive subsistence practices [60,61]. In terms of animals, a higher ratio of caprines to pigs and cattle might suggest more extensive pastoral subsistence practices, while a lower ratio would signal the practice focused on more intensive agricultural activities [61]. We applied this perspective to the sites mentioned in this study. Due to the absence of archaeobotanical data from Yucun, our focus was primarily on animal data. Our findings suggest discernable differences between the sites in the Longshan Mountain region and those in the middle and lower Yellow River valley, in the taxonomic composition of domestic animals, particularly in terms of the ratio of caprines to pigs and cattle (Table 3). Overall, the sites in the Longshan Mountain region exhibited higher ratios of caprines to pigs and cattle (0.38–0.53 by NISP, 0.47–0.88 by MNI), whereas lower ratios were recorded for the sites in the middle and lower reaches of the Yellow River (0.01–0.39 by NISP, 0.06–0.44 by MNI). This implies that intensive agricultural activities seemingly played a more important role in the subsistence economies of residents in the middle and lower Yellow River valley compared to their counterparts in the Longshan Mountain region. This contrast is particularly evident at ZGGC and WCG, where large walled cities dating back to the first millennium BC have been discovered. However, TM-QC appears to be an exception, showing a ratio similar to the sites in the Longshan Mountain region.

**Figure 9:**
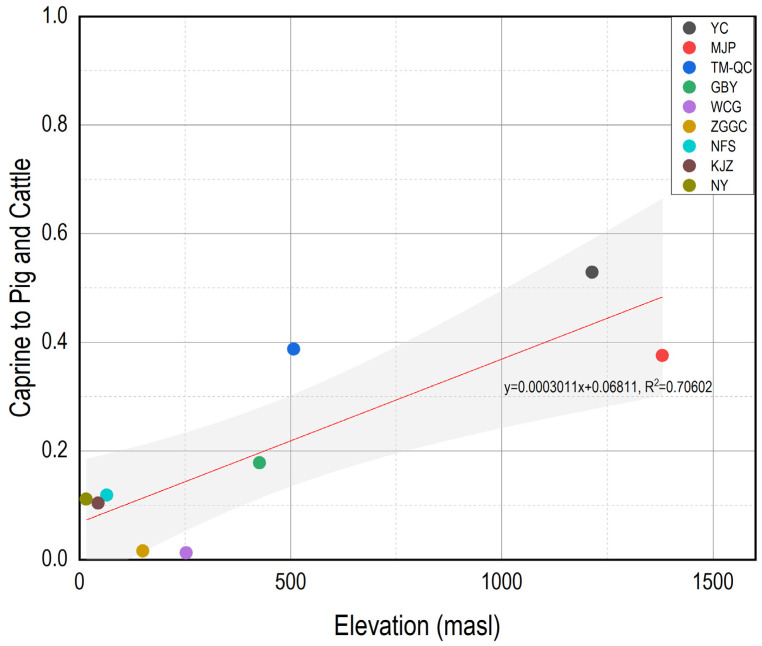
Scatterplot of elevation and the ratio of caprine to pig and cattle (by NISP) for each site.

The similarities and differences observed among these sites may be attributed to their respective elevations. In fact, MJP, XS, and TM-QC share a relatively similar topography, situated either on the Loess Plateau or its edges, all exceeding an elevation of 500 m above sea level (masl). In contrast, the remaining sites are all located on flatter terrains at significantly lower elevations. It appears that the strategies for domestic animal use were somewhat influenced by the elevation (Figure 9). A Pearson’s correlation test demonstrated a positive correlation between the site elevation and the ratio of caprines to pigs and cattle in terms of both the NISP and MNI (NISP: *p* < 0.01, r = 0.840; MNI: *p* < 0.05, r = 0.756). The residents’ preference for cattle and caprine herding at higher elevations could be linked to their adaptation to cooler and drier environmental conditions in the Longshan Mountain region. Sheep, goats, and cattle were initially domesticated in West Asia at ca. eighth millennium BC [62,63] and later spread into Central Asia by ca. sixth millennium BC [64]. These domestic animals appeared in the Gansu-Qinghai region and the northern Loess Plateau region of China during the third to second millennium BC [65,66,67]. The geographic and environmental conditions in the Longshan Mountain region likely facilitated the development of caprine and cattle herding.

## 5. Conclusions

The analysis of the animal remains from Yucun provides important insights into the subsistence practices in relation to animal resource exploitation among the individuals associated with the Zhou culture in the first half of the first millennium BC. Our findings indicate that cattle, caprines, and pigs were the primary livestock raised by the residents at Yucun, mainly for meat production. A comparative analysis between Yucun and the Qin cultural sites, Maojiaping and Xishan, reveals consistency in the composition of domestic animals in the western and eastern areas of the Longshan Mountain region. During the first millennium BC, the Longshan Mountain region exhibited distinct animal resource exploitation practices compared to those in the middle and lower reaches of the Yellow River valley. This suggests that environmental conditions may have played a more significant role than cultural factors in shaping the strategies for animal resource exploitation within the region. Our datasets from Yucun add depth into the ongoing discourse on animal husbandry, landscape use across cultures, and environmental conditions. This, in turn, facilitates a better understanding of the human–land interactions in northern China during the first millennium BC.

## Figures and Tables

**Table 1:** The NISP and MNI of animal remains from Yucun.

Category	Taxon	NISP	NISP%	MNI	MNI%
Domestic mammals					
Carnivora	Dog, *Canis familiaris*	365	8.1%	35	14.4%
Perissodactyla	Horse, *Equus caballus*	464	10.3%	16	6.6%
Artiodactyla	Pig, *Sus domesticus*	917	20.3%	45	18.5%
	Cattle, *Bos taurus*	1225	27.1%	41	16.9%
	Sheep, *Ovis aries*	73	1.6%	13	5.3%
	Goat, *Capra hircus*	80	1.8%	10	4.1%
	Sheep/goat, *Ovis aries/Capra hircus*	981	21.7%	17	7.0%
Total domestic mammals		4105	90.8%	177	72.8%
Wild mammals					
Erinaceomorpha	Hedgehog, *Erinaceus europaeus*	1	0.0%	1	0.4%
Lagomorpha	Hare, *Lepus* sp.	24	0.5%	4	1.6%
Rodentia	Zokor, *Myospalax* sp.	1	0.0%	1	0.4%
	Chinese bamboo rat, *Rhizomys sinensis*	2	0.0%	1	0.4%
	Unidentified rodents	20	0.4%	5	2.1%
Carnivora	Wolf, *Canis Lupus*	1	0.0%	1	0.4%
	Fox, *Vulpes* sp.	1	0.0%	1	0.4%
	Black bear, *Ursus thibetanus*	2	0.0%	1	0.4%
	Tiger, *Panthera tigris*	1	0.0%	1	0.4%
	Cat, *Felis* sp.	2	0.0%	1	0.4%
Artiodactyla	Wild pig, *Sus scrofa*	2	0.0%	1	0.4%
	Sika deer, *Cervus nippon*	109	2.4%	10	4.1%
	Roe deer, *Capreolus capreolus*	59	1.3%	7	2.9%
	Large cervid	35	0.8%	5	2.1%
	Small cervid	8	0.2%	2	0.8%
	Large artiodactyl	5	0.1%	1	0.4%
Total wild mammals		273	6.0%	43	17.7%
Molluscs					
Lamellibranchia	Unio douglasiae, *Unio douglasiae*	24	0.5%	5	2.1%
	Unionidae	12	0.3%	2	0.8%
Fishes					
Osteichthyes	Unidentified species	4	0.1%	1	0.4%
Reptiles					
Trionychidae	Soft shell turtle, Trionychidae	12	0.3%	3	1.2%
Emydidae	Freshwater turtle, Emydidae	6	0.1%	1	0.4%
Aves					
Falconiformes	Accipitridae	1	0.0%	1	0.4%
Galliformes	Unidentified phasianid, *Phasianus* sp.	58	1.3%	6	2.5%
	Large bird	1	0.0%	1	0.4%
	Medium bird	22	0.5%	2	0.8%
	Small bird	3	0.1%	1	0.4%
Total wild non-mammals		143	3.2%	23	9.5%
Total		4521	100.0%	243	100.0%

**Table 2:** Bone epiphyseal fusion data and age profile for cattle from Yucun.

	7–10 Months	12–18 Months	24–36 Months	36–48 Months
Fused and Fusing No.	62	171	50	33
Percentage	100.0%	98.3%	79.4%	70.2%
Unfused No.	0	3	13	14
Percentage	0.0%	1.7%	20.6%	29.8%

**Table 3:** The MNI of pigs, cattle, and caprines, and the ratio of caprines to pigs and cattle from archaeological sites of the first millennium BC in the Yellow River valley.

Site	Quantification	Caprines	Pigs	Cattle	Ratio of Caprines to Pigs and Cattle
Yucun	NISP	1134	917	1225	0.53
	MNI	40	45	41	0.47
MJP	NISP	188	348	152	0.38
	MNI	14	10	6	0.88
XS	NISP	/	/	/	/
	MNI	6	10	5	0.40
GBY	NISP	18	65	36	0.18
	MNI	1	3	2	0.20
TM-QC	NISP	650	608	1068	0.39
	MNI	43	75	22	0.44
WCG	NISP	14	1039	63	0.01
	MNI	1	13	2	0.07
NFS	NISP	7	48	11	0.12
	MNI	2	7	3	0.20
NY	NISP	10	39	51	0.11
	MNI	2	14	8	0.09
ZGGC	NISP	37	1652	592	0.02
	MNI	5	69	14	0.06
KJZ	NISP	49	323	147	0.10
	MNI	7	20	8	0.25

## Data Availability

All relevant data are included in this manuscript.
